# The role of DNA methylation in human pancreatic neuroendocrine tumours

**DOI:** 10.1530/EO-23-0003

**Published:** 2023-04-17

**Authors:** Katherine A English, Rajesh V Thakker, Kate E Lines

**Affiliations:** 1 OCDEM, Radcliffe Department of Medicine, University of Oxford, Churchill Hospital, Oxford, UK; 2 Oxford NIHR Biomedical Research Centre, Oxford University Hospitals Trust, Oxford, UK

**Keywords:** epigenetics, methylation, multiple endocrine neoplasia type 1, menin, pancreatic neuroendocrine tumours, hydroxymethylation

## Abstract

Pancreatic neuroendocrine tumours (PNETs) are the second most common pancreatic tumour. However, relatively little is known about their tumourigenic drivers, other than mutations involving the multiple endocrine neoplasia 1 (*MEN1)*, ATRX chromatin remodeler, and death domain-associated protein genes, which are found in ~40% of sporadic PNETs. PNETs have a low mutational burden, thereby suggesting that other factors likely contribute to their development, including epigenetic regulators. One such epigenetic process, DNA methylation, silences gene transcription *via* 5’methylcytosine (5mC), and this is usually facilitated by DNA methyltransferase enzymes at CpG-rich areas around gene promoters. However, 5’hydroxymethylcytosine, which is the first epigenetic mark during cytosine demethylation, and opposes the function of 5mC, is associated with gene transcription, although the significance of this remains unknown, as it is indistinguishable from 5mC when conventional bisulfite conversion techniques are solely used. Advances in array-based technologies have facilitated the investigation of PNET methylomes and enabled PNETs to be clustered by methylome signatures, which has assisted in prognosis and discovery of new aberrantly regulated genes contributing to tumourigenesis. This review will discuss the biology of DNA methylation, its role in PNET development, and impact on prognostication and discovery of epigenome-targeted therapies.

## Introduction

Pancreatic neuroendocrine tumours (PNETs) account for approximately 10% of all pancreatic tumours and occur with an incidence of approximately 1 per 100,000 (Sonbol *et al.* 2022). The incidence of PNETs is rising, partly due to increased detection rates and improved histopathological diagnosis. Compared to other malignancies, PNETs tend to be well-differentiated indolent tumours, with >15% caused by germline mutations in the multiple endocrine neoplasia 1 (*MEN1*), Von Hippel–Lindau tumour-suppressor (*VHL*), TSC complex (*TSC*)*,* neurofibromin 1(*NF1*), MutY DNA glycosylase, BRCA2 DNA repair-associated, cyclin-dependent kinase inhibitor 1B (*CDKN1B*), and checkpoint kinase 2 genes (Crona & Skogseid 2016, Scarpa *et al.* 2017). Although germline mutations in these genes have been found in patients with clinically sporadic PNETs, these germline mutations are not always associated with a PNET phenotype. PNETs associated with hereditary tumour syndromes occur most commonly in patients with MEN1 syndrome (~80%), followed by VHL (5–17%) and TSC (4%) (de Laat *et al.* 2016, Romanet *et al.* 2019, Ahmad *et al.* 2021, Evans *et al.* 2022*b*). Separate from PNETs are poorly differentiated highly aggressive pancreatic neuroendocrine carcinomas (PNECs). Although these pancreatic cancers retain and/or express neuroendocrine features, they are biologically and clinically distinct from their indolent PNET counterparts. Four distinct pathways have been implicated in PNET pathogenesis, including chromatin remodelling, DNA damage repair, mammalian target of rapamycin (mTOR) pathway activation, and telomere maintenance (Scarpa *et al.* 2017). In sporadic (i.e. non-familial) PNETs, inactivating mutations in *MEN1*, alpha thalassemia/mental retardation syndrome x-linked (ATRX) chromatin remodeller (*ATRX*), and death domain-associated protein (*DAXX*) are the most common, occurring in up to 40% of PNETs, and are involved in epigenetic regulation (Jiao *et al.* 2011, Thakker 2014, Chan *et al.* 2018) and inhibition of proliferative pathways (Jiao *et al.* 2011, Chamberlain *et al.* 2014). For example, menin (encoded by *MEN1*) is a ubiquitously expressed protein which forms complexes with proteins involved in gene transcription and repression mainly *via* histone modifications, including histone 3, lysine 4 (H3K4) and H3K9 methylation, and histone deacetylation (HDAC) (Yang *et al.* 2013, Thakker 2014). In addition to these genetic mutations, the tumour-suppressor protein RASSF1A is silenced in >80% of PNETs due to increased DNA methylation at its promoter. Given the low mutational burden of PNETs and that genes most commonly mutated in PNETs are involved in epigenetic regulation, recent studies have focused on the investigation of the epigenome, including both histone modifications and DNA methylation. Histone modification and DNA methylation mechanisms are intrinsically linked; however, recently new technological and scientific developments have advanced our understanding of DNA methylation. This review will focus on the biology of DNA methylation, its role in human PNET development, and its likely impact on current therapeutics and future research.

## Epigenetics overview

**Figure 1:**
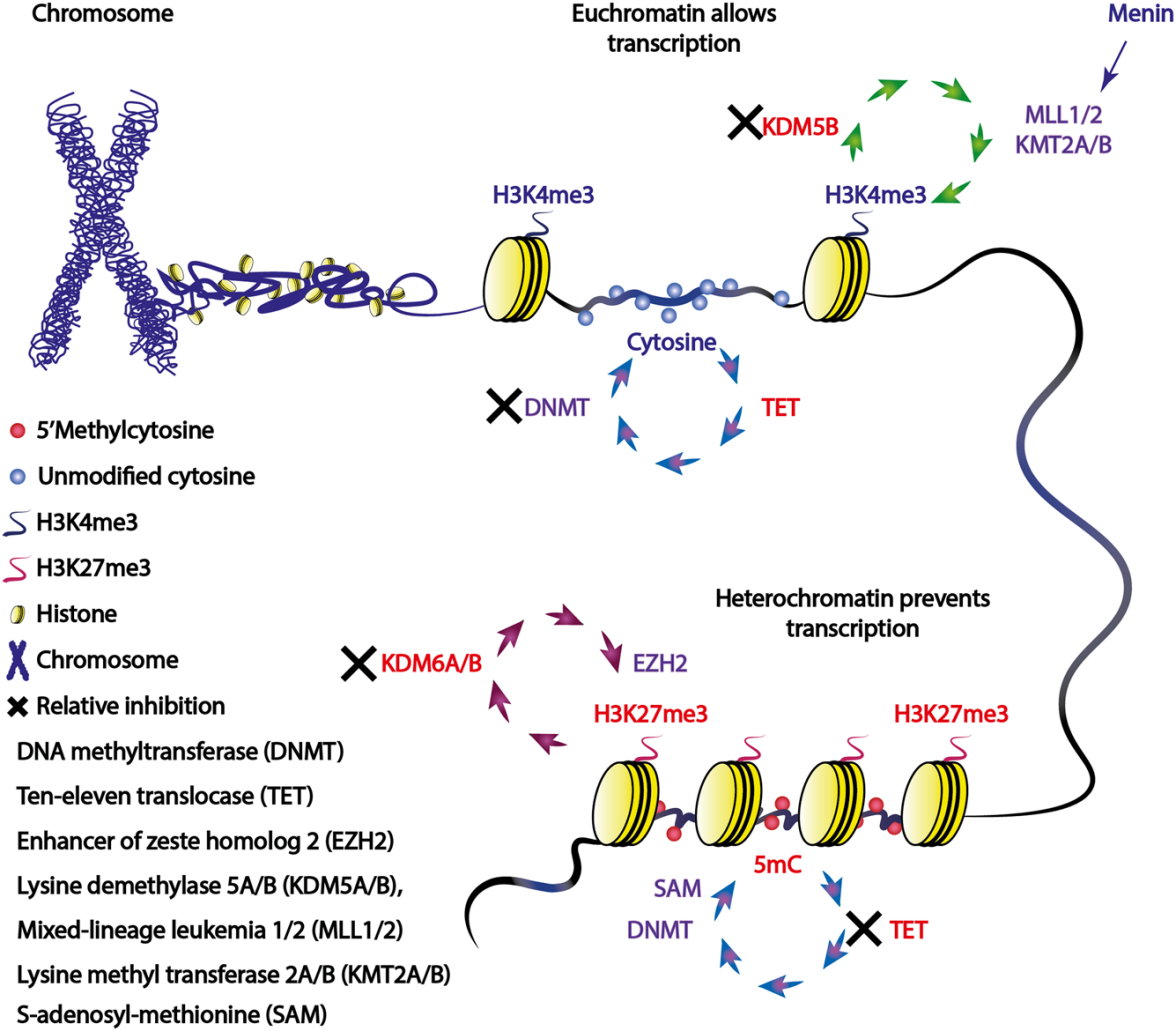
Relationship between histone and DNA methylation with chromatin state. In chromosomes, DNA is usually tightly wrapped and packaged around histone proteins when not being actively transcribed. DNA methylation is catalysed by DNMT enzymes which ensure that cytosines at CpG sites remain methylated and this prevents transcriptional machinery from binding to these sections of DNA. Histone and DNA methylation work together to either allow or prevent DNA transcription. Thus, sections of DNA are ‘marked’ for transcription with both histone and DNA modifications to determine which parts of DNA are unwound from histone proteins to enable transcriptional machinery to access DNA. TET enzymes ensure that DNA remains unmethylated, thereby allowing transcription factors to bind to DNA, whereas the methylation mark H3K27 tri-methylation (H3K27me3), catalysed by EZH2, is associated with heterochromatin and keeps DNA wound tightly around histone proteins. Menin catalyses the addition of a methyl group by MLL1/2 (KMT2A/B) to form the active histone methylation mark H3K4 tri-methylation (H3K4me3), which unwinds DNA.

Epigenetics refers to processes that alter gene activity without changing the DNA sequence and result in modifications that may be transmitted to daughter cells. These epigenetic processes, which include methylation, acetylation, phosphorylation, ubiquitination, and sumoylation, have important roles in ensuring cell-specific transcription regulating the accessibility of DNA, as follows. DNA, whose total length in a human cell is >2 m, is packaged within the nucleus, whose diameter is ~10–20 μm, by being tightly wrapped around histone proteins to form nucleosomes that are the building blocks for chromatin (Fig. 1) (Annunziato 2008). Chromatin may occur in a less tightly compacted form, referred to as open or euchromatin, which is closely associated with RNA polymerases and actively transcribed genes, while more condensed chromatin, referred to as closed or heterochromatin, is associated with structural proteins and regions containing inactive genes. DNA methylation and histone modifications, both of which determine chromatin state, are dynamic processes, and these may also change depending on the microenvironment and nutrient availability (Tobi *et al.* 2009).

The histone mark that is added to specific amino acids on histone tails determines the chromatin structure. For example, the tumour-suppressor protein menin forms complexes with mixed-lineage leukemia gene 1/2 (*MLL1/2*)/lysine methyltransferase 2A/B (*KMT2A/B*), which adds a methyl group to lysine 4 of histone protein H3 (Fig. 1), forming the active histone mark H3K4 tri-methylation (H3K4me3) and open chromatin. Whereas tri-methylation of lysine 27 of histone 3 (H3K27me3), catalysed by enhancer of zeste homolg 2 (*EZH2*), leads to a closed chromatin (heterochromatin) state and subsequent transcriptional repression. Both *MLL1/2 (KMT2A/B)* and *EZH2* are known are ‘writers’ as they are responsible for adding these marks, whereas lysine demethylase 5B (*KDM5B*) and lysine demethylase 6A/B (*KDM6A/B*) are demethylases, which remove these methyl groups, and are termed ‘erasers’ (Table 1).

**Table 1:** Examples of methylation-associated histone H3 and DNA epigenetic ‘writers’, ‘readers’, and ‘erasers’.

Epigenetic mark	Transcription	Writers	Readers	Erasers
**Histones**				
H3K4 methylation	Active	KMT2A/B (MLL1/2)	TAF3 Sgf29	KDM5A/B (JARID1A/B)
		SETD1A/B (KMT2F/E)	CHD1 BPTF	KDM1A/B (LSD1/2)
H3K27 methylation	Repressive	EZH1/2 (KMT6A/B)	CBX7 BAHD1	KDM6A/B
DNA				
5mC	Repressive	DNMT1, DNMT3A, DNMT3B	MeCP2 MBD1–6 Kaiso family SRA family	TET1-3

5mc, 5’methylcytosine; BAHD1, bromo adjacent homology domain containing 1; BPTF, bromodomain finger transcription factor; CDX7, chromobox 7; CHD1, chromodomain helicase DNA-binding protein 1; DNMT1/3A/3B, DNA methyltransferase 1/3A/3B; EZH1/2, enhancer of zeste 1/2 polycomb repressive complex 2 subunit; JARID1A/B, Jumonji, AT-rich interactive domain 1A/B; KDM1A/B, lysine demethylase 1A/B; KDM5A/B, lysine demethylase 5A/B; KDM6A/B, lysine demethylase 6A/B; KMT2A/B, lysine methyltransferase 2A/B; KMT2E/F, lysine *N*-methyltransferase 2E/F; KMT6A/B, hstone–lysine *N*-methyltransferase EZH1/2; LSD1/2, lysine-specific histone demethylase 1A/B; MBD1–6, methyl–CpG-binding domain protein 1–6; MeCP2, methyl–CpG-binding protein 2; MLL1/2, myeloid/lympoid or mixed-lineage leukaemia; Sgf29, SAGA complex-associated factor 29; SRA, SET and ring-finger-associated; SETD1A/B, SET domain containing 1A/B, histone lysine methyltransferase; TAF3, TATA-box-binding protein-associated factor 3; TET1–3, tet methylcytosine dioxygenase 1–3.

**Figure 2:**
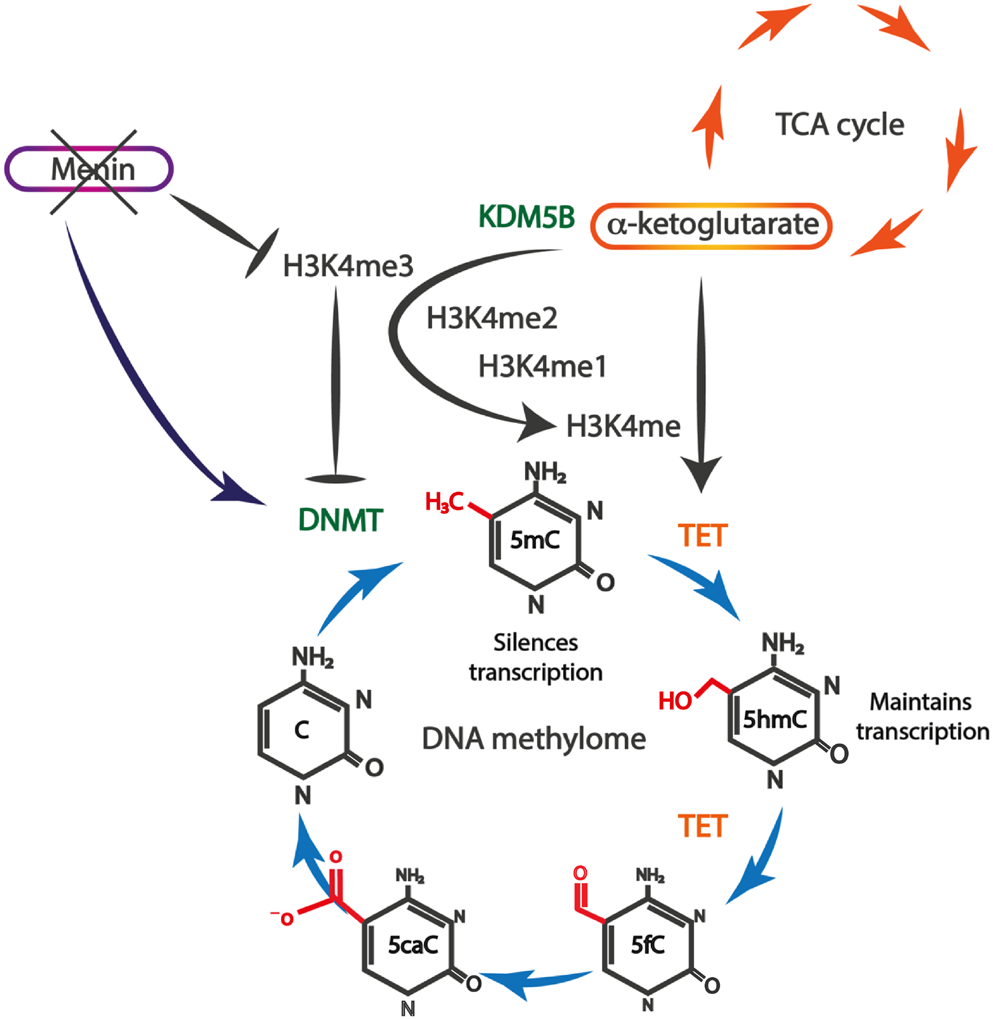
The dynamic DNA methylome cycle. In the dynamic DNA methylome, 5’methylcytosine (5mC) undergoes consecutive oxidative steps to form 5’hydroxymethylcytosine (5hmC), 5’formylcytosine (5fC) and 5’carboxylcytosine (5caC) and then back to an unmodified cytosine (C), which can re-enter the cycle following re-methylation by DNA Methyltransferase (DNMT) enzymes to 5mC. The DNA methylome is linked with the tricarboxylic acid (TCA) cycle, which is also known as the Krebs or citric acid cycle. The TCA cycle provides alpha-ketoglutarate which is required for active demethylation by ten-eleven-translocase (TET) and by histone lysine demethylase (KDM) enzymes including KDM5B which demethylates H3K4me3, H3K4 dimethylation (H3K4me2), and H3K4 mono-methylation (H3K4me1). Loss of menin leads to increased DNMT1 and subsequent DNA methylation, as well as a loss of the active histone mark H3K4me3, which also protects against DNA methylation.

**Figure 3:**
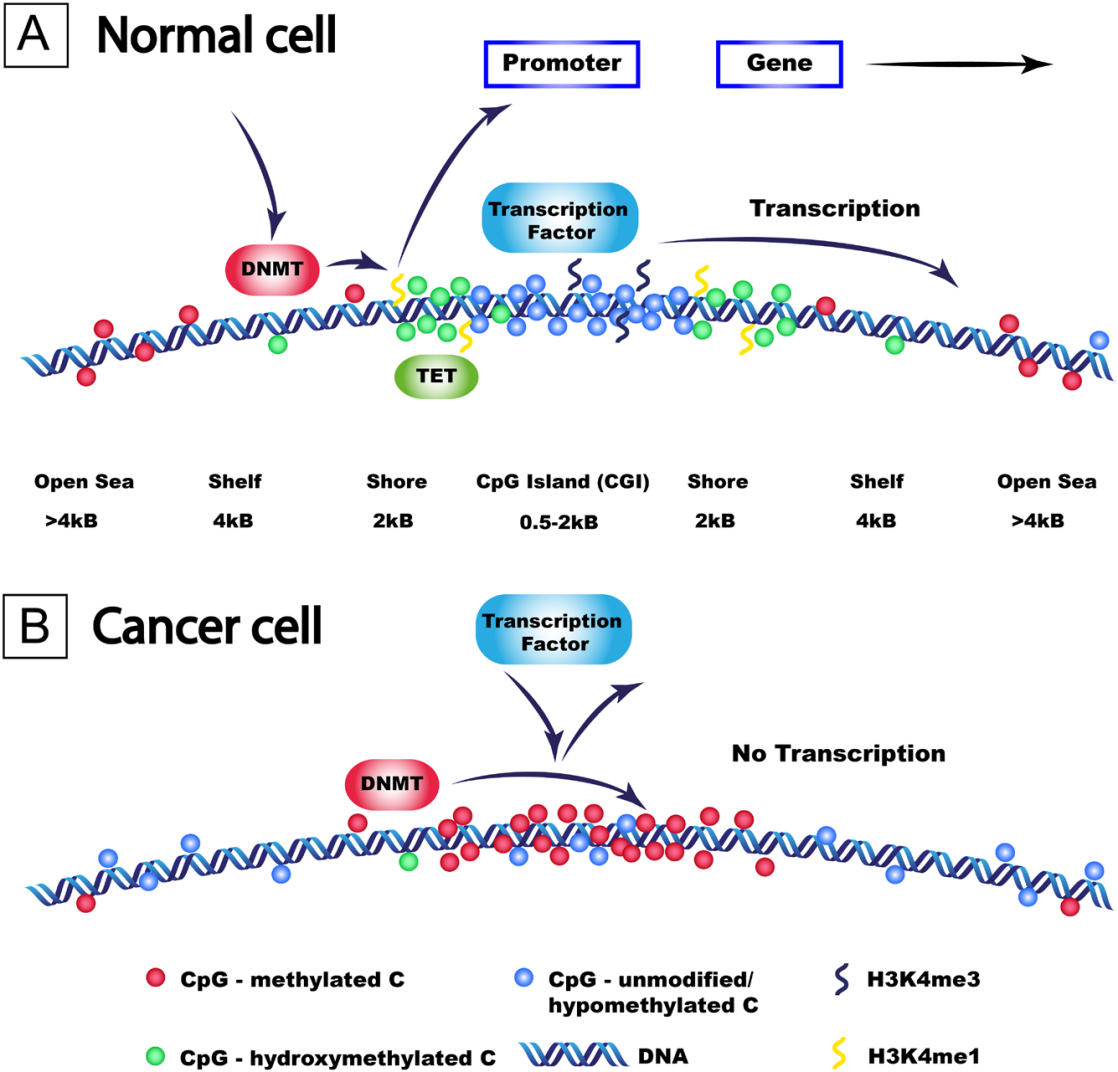
DNA methylation in normal (panel A) and cancer (panel B) states. (A) A typical strand of DNA with a CpG island I (CGI) in normal tissue. CGIs are flanked on either side by shores (within 2 kB of the CGI), shelves (within 4 kB), and the open sea (>4kB). CpG sites occur more frequently in CpG islands when compared to the rest of the genome and are usually hypomethylated (blue circles), whilst 5’hydroxymethylcytosine (5hmC) marks (green circles) tend to be present at the shores of CGI and protect against DNA methyltransferases (DNMTs), and subsequent 5’methylcytosine (5mC) marks (red circles) are found less frequently outside of CGIs. H3K4 tri-methylation (H3K4me3) marks are associated with regions of DNA hypomethylation and H3K4 mono-methylation (H3K4me1) marks are associated with regions enriched in 5hmC. CpG sites in the open sea (i.e. >4 kB away from a CGI) tend to be methylated. (B) In cancer, aberrant DNA methylation occurs with a loss of 5hmC marks (green circles) that results in an inability to protect against DNMTs, which leads to the usually hypomethylated cytosines (blue circles) in CGI becoming methylated (red circles) by DNMTs that in turn leads to transcriptional silencing. Scattered CpGs outside the CGI (shelves and open sea) become progressively hypomethylated in malignancy.

‘Readers’ are proteins which decode these histone marks and determine the recruitment of other machinery to assist in changing DNA conformation to either allow or inhibit transcription. There are >75 different ‘writers’, ‘erasers’, and ‘readers’ involved in methylation maintenance of histone H3, and examples of these are provided in Table 1 (Hyun *et al.* 2017, Beacon *et al.* 2021). Separate to these histone marks are DNA modifications, with DNA methylation being the most common and characterised mark. Long-term gene silencing may occur *via* DNA methylation, with the modified DNA base 5’methylcytosine (5mC) responsible for recruiting transcriptional repressors to DNA protomers, and/or inhibiting transcriptional factor binding, ultimately silencing gene expression (Kohli & Zhang 2013, Moore *et al.* 2013). Cytosine modifications are recognised by different transcription factors, which show a preference for specific cytosine modifications, including the methyl-binding domain (MBD), Kaiso, and SET- and ring-finger-associated (SRA) domain family (Ren *et al.* 2018). There are several ‘reader’ proteins which can interact with 5mC, and these predominantly contain an MBD domain, including MBD1–6 and methyl–CpG-binding protein 2 (MeCP2), SET domain bifurcated histone lysine methyltransferase 1/2, and bromodomain adjacent to zinc finger domain 2A/B. By interacting with 5mC, these proteins predominantly cause transcriptional repression, either by recruitment of other transcriptional repressive proteins or by direct interaction with histone modifications (e.g. MeCP2 which binds to histone ‘writers’ and ‘erasers’, which are involved in HDAC and histone methylation, respectively) that subsequently alter nucleosome and chromatin structure to a closed state (i.e. heterochromatin) (Du *et al.* 2015). 5mC is formed by DNA methyltransferase (DNMT) enzymes transferring a methyl group from S-adenosyl-methionine to the 5’ position of cytosine (Fig. 1) (Moore *et al.* 2013). There are three human DNMTs: *DNMT3A* and *DNMT3B* are responsible for *de novo* methylation and *DNMT1* is responsible for methylation maintenance during replication. The function of these DNMTs is directly opposed by the recently discovered ten-eleven-translocase (TET) family of enzymes, *TET1*, *TET2* and *TET3*, which can actively demethylate 5mC *via* consecutive reactions from 5mC back to an unmodified/hypomethylated cytosine (Tahiliani *et al.* 2009, Ito *et al.* 2010, Kohli & Zhang 2013). The DNA methylome is therefore a dynamic process that is also closely intertwined with the citric acid cycle, with TET and a subset of lysine demethylases (KDMs) dependent on alpha-ketoglutarate, including KDM5B which demethylates H3K4me3, H3K4 di-methylation (H3K4me2), and H3K4 mono-methylation (H3K4me1) histone marks (Fig. 2). 5’hydroxymethylcytosine (5hmC; the first intermediate mark during 5mC oxidation) is a stable epigenetic mark, protecting CpG sites against DNMTs forming 5mC and promoting gene transcription (Kohli & Zhang 2013, Skvortsova *et al.* 2019). However, 5hmC and 5mC are indistinguishable when using conventional bisulfite conversion techniques, and this may explain the reported inconsistencies between apparently methylated promoters and protein expression in PNETs (e.g. *MEN1* andO-6-methylguanine-DNA methyltransferase (*MGMT*)) and in PNECs (SRY-box transcription factor 2 (*SOX2)*) (Arnold *et al.* 2007, Walter *et al.* 2015, Ban *et al.* 2022, Yachida *et al.* 2022). For example, SOX2 overexpression was reported in PNECs to be associated with promoter methylation, and a paradoxically open chromatin structure at the *SOX2* gene was observed using assay for transposase-accessible chromatin with high-throughput sequencing (Yachida *et al.* 2022). 5mC and 5hmC occur almost exclusively at sites where cytosine is followed by a guanine (CpG) on cis-DNA. Either CpGs are heavily methylated and scattered at lower than expected frequency throughout the human genome which is likely due to 5mC undergoing spontaneous deamination to thiamine (Bird 1986) or they are found in clusters of hypomethylated CpGs in sections of DNA 0.5–2kB in length, termed CpG islands (CGIs). There are approximately 30,000 CGIs which are commonly found in proximal promoters and specifically those of housekeeper genes (Bird 1986, Jones & Baylin 2002). CGIs are flanked on either side by shores (within 2kB of the CGI), shelves (within 4 kB), and the open sea (>4 kB) (Fig. 3). When genes are transcribed, CpG sites within the CGI are hypomethylated and flanked on either side by 5hmC at shores (which protect against 5mC), with 5mC marks scattered throughout the open sea (Fig. 3A). 5hmC marks are also present at the ‘rim’ of expressed genes, with the amount of 5hmC positively correlating with both the peak in H3K4me3 histone marks and with gene expression (Li *et al.* 2018). In cancer cells, when 5hmC is lost (Fig. 3B), DNMTs methylate these previously protected unmethylated cytosines in CGIs, leading to 5mC formation and transcriptional silencing. Aberrant DNA hypermethylation tends to occur at hypomethylated CGIs including tumour-suppressor genes (TSGs) (Skvortsova *et al.* 2019), whereas overall DNA hypomethylation commonly occurs outside of CGIs at highly repetitive DNA sequences, mainly at short or long interspersed nuclear elements (SINEs or LINEs) which comprise up to 50% of the human genome, and is associated with chromosome instability (Eden *et al.* 2003, Ehrlich 2009).

## DNA methylation in PNETs

There are many methods to investigate DNA methylation, and these include bisulfite-based, restriction enzyme-based, and affinity-based strategies (Olkhov-Mitsel & Bapat 2012). There have been 32 studies reporting DNA methylation in human PNETs, and one study which has looked at global 5hmC (Tables 2 and 3). The PNET methylome has been profiled in 24 studies in a gene-specific manner (Muscarella *et al.* 1998, Bartsch *et al.* 2000, Serrano *et al.* 2000, Chan *et al.* 2003, Dammann *et al.* 2003, House *et al.* 2003, Wild *et al.* 2003, Liu *et al.* 2005, Arnold *et al.* 2007, Choi *et al.* 2007, Dejeux *et al.* 2009, Malpeli *et al.* 2011, Stricker *et al.* 2012, Ohki *et al.* 2014, Schmitt *et al.* 2014, Stefanoli *et al.* 2014, Liu *et al.* 2014, Cros *et al.* 2016, Ushiku *et al.* 2016, Campana *et al.* 2018, Conemans *et al.* 2018, Zhang *et al.* 2020, Ban *et al.* 2022, Evans *et al.* 2022*a*) and subsequently by both global methylation (Marinoni *et al.* 2017) and hydroxymethylation (Sharma *et al.* 2022) and by specific CpG site assessment with array-based technologies (Chan *et al.* 2003, Tirosh *et al.* 2019, Boons *et al.* 2020, Di Domenico *et al.* 2020, Lakis *et al.* 2021, Simon *et al.* 2022, Yachida *et al.* 2022). Out of the 24 studies looking at a specific subset of genes, 58% (14/24) have used methylation-specific polymerase chain reaction (MSP) to investigate the percentage of methylation present at specific gene promoters. However, the criteria used to classify whether a gene is methylated were not defined in most studies. Definitions for methylated genes, if included, were reported as increased gene methylation as an mCG/CG ratio of >7%, >8, or >20% (Malpeli *et al.* 2011, Cros *et al.* 2016, Campana *et al.* 2018, Li *et al.* 2018). Other techniques include combined bisulfite restriction analysis (COBRA), methylation-specific multiplex ligation-dependent probe amplification (MS-MLPA), denaturing HPLC, pyrosequencing, and array-based techniques including Illumina Infinium Human450K and MethylationEPIC arrays. All studies examining DNA methylation have used bisulfite-only methods to investigate the PNET methylome. However, bisulfite converts only unmodified cytosines to uracil (subsequently to thiamine), and it is important to note that both 5mC and 5hmC marks will remain unchanged and will therefore be indistinguishable.

**Table 2:** Gene-specific DNA methylation studies of human PNETs.

Study	PNETs	Investigation	Genes investigated (proportion methylated (%))	Other features
**Gene-specific methylation assessment grouped by method**				
Arnold *et al.* (2007)	46 PNETs: 26 INS, 3 GLU, 4 GAS, 2 VIP, 11 NF	MSP, IHC, LOH, MSI	*APC* (48%), E-cadherin (2%), *HIC-1* (93%), *hMLH1* (0%), *MEN1* (19%), *MGMT* (17%), *p16* (0%), *PTEN* (0%), *RASSF1A* (80%), *RUNX3* (7%), and *TIMP3* (0%)	CIMP-positive phenotype associated with a higher Ki67 and poorer overall survival. APC, p16, and menin expression for each tumour was reported using IHC
Bartsch *et al.* (2000)	17 PNETs: 17 INS	MSP	*CDKN2A* (17%)	
Campana *et al.* (2018)	43 PNETs	MSP or PSQ	*MGMT*	On multivariate analysis, poorer overall survival was associated with unmethylated *MGMT*, higher Ki67, and previous chemotherapy
Chan *et al.* (2003)	11 PNETs: 1 INS, 2 GAS (2 MEN1), 8 NF (1 MEN1)	MSP and COBRA	*CACNA1G* (0%), *COX2* (9%), *ER* (64%), *MEN1* (0%), *MGMT* (0%), *APBA1/MINT1* (18%), *APBA2/MINT2* (0%), *MINT25* (0%), *MINT27* (9%), *MINT31* (18%), *P14* (9%), *P16* (9%), *RARß* (0%), *THBS1* (9%)	Percentage of methylated genes in adjacent normal tissue: *COX2* (9%), *ER* (27%), *MINT1* (27%), *MINT27* (18%), *MINT31* (9%)
Dammann *et al.* (2003)	12 PNETs	MSP	*CDKN2A* (17%), *RASSF1A* (83%)	*CDKN2A* and *RASSF1A* methylation was associated with metastatic disease
House *et al.* (2003)	48 PNETs: 1 GLU, 2 GAS, 45 NF	MSP	*APC* (21%), *CDKN2A* (40%), E-cadherin (23%), *GST* (0%), *hMLH1* (23%), *MGMT* (40%), *P14* (0%), *P73* (17%), *RARß* (25%), *RASSF1A* (75%), *TIMP3* (0%)	*CDKN2A* methylation was associated with mortality (multivariate analysis)
Liu *et al.* (2005)	16 PNETs	MSP	*MGMT* (13%), *P14* (44%), *P16* (19%), *RASSF1A* (63%)	Percentage of methylated genes in adjacent normal tissue: *RASSF1A* (27%)
Malpeli *et al.* (2001)	20 PNETs: 3 INS, 2 GAS, 15 NF	MSP, qMSP, mRNA	*RASSF1A* (80%), RASSF1A (55%)	RASSF1A expression was increased after treatment of BON-1, QGP-1, and CM cells
Muscarella *et al.* (1998)	4 PNETs: 3 NF, 1 liver metastasis	MSP	*CDKN2A* (50%)	50% homozygous loss of CDKN2A. No genetic mutations in *CDKN2A* were identified
Ohki *et al.* (2014)	50 PNETs: 2 INS, 48 NF	MSP, LOH, mRNA	*PHLDA3* (82%) – analysed in 11 patient PNET samples only	*PHLDA3* methylation was associated with poorer outcomes (ns). A99 cell lines treated with 5-aza-C decreased methylation of PHLDA3
Schmitt *et al.* (2014)	52 PNETs: 28 INS, 3 GLU, 5 GAS, 4 VIP, 12 NF	MSP, IHC	*MGMT* (56%)	MGMT loss of expression correlated with an overall poorer survival. 51% of samples had discordant promoter methylation and protein expression
Serrano *et al.* (2000)	9 PNETS: 9 GAS	MSP and semi-Q-MSP	*CDKN2A/P16* (67%)	No genetic mutations or homozygous deletions. No association with clinical characteristics
Ushiku *et al.* (2016)	36 PNETs: 6 INS, 2 GAS, 1 VIP, 1 SOM, 26 NF	Q-MSP, IHC	*HOPX* (14%)	HOPX reduced expression associated with poorer overall survival
Wild *et al.* (2003)	21 PNETs: 5 INS, 5 GAS, 1 VIP, 1 REN, 9 NF	MSP, RNA, and IHC	*TIMP3* (44%)	*TIMP3* methylation was associated with metastasis. Strong TIMP3 expression was seen in normal islets, with 55% of PNETs showing loss of expression
Ban *et al.* (2022)	115 PNETs: 44 NF and 71 functional	MS-MLPA (17 PNETs and 7 normal pancreata) and IHC	*MLH1, MSH2*, *MSH6*, *PMS2* (23.5% had promoter methylation in at least 1 of the MMR genes), *MGMT* (47%)	All samples expressed MMR proteins. Multivariate analysis, low MGMT expression was associated with shorter progression-free survival
Conemans *et al.* (2018)	95 PNETs: NF (61 MEN1, 34 sporadic)	SALSA MS-MLPA	*APAF1*, *APC*, *BCL2*, *CASP8*, *CD44*, *CDH13*, *CKDN2B*,* DNAJC15*, *ESR1*, *GATA4*, *GATA5*, *GSTP1*, *KLLN, MGMT*, *MSH6*, *MUS81*, *NF1*, *NTRK1*, *PAX5*, *PCCA*, *RARRES1*, *RASSF1*, *SFRP1*, *TERT*, *THBS1*, *TP73*, *TWIST1* ^a^	Using a cutoff level of 15% to define promoter hypermethylation, *MSH6*, *APAF1*, *RASSF1*, *TWIST1,* and *KLLN* were hypermethylated in >80% of MEN1 tumours. Of note, *RASSF1* and *CASP8* had high levels of promoter methylation in menin-negative tumours compared to menin-positive tumours
Stefanoli *et al.* (2014)	56 PNETs: 23 INS, 2 GLU, 2 GAS, 2 VIP, 1 CUS, 26 NF	MS-MLPA and CNA	*ATM*, *APC*, *BRCA1*, *BRCA2*, *CADM1*, *CASP8*, *CDH13*, *CD44*, *CDKN1B*, *CDKN2A(p14 and p16)*, *CHFR*, *DAPK1*, *ESR1*, *FHIT*, *GATA5*, *GSTP1*, *HIC1*, *LINE-1*, MGMT, *MLH1*, *PAX5*, *PAX6*, *PYCARD*, *RARß*, *RASSF1*, *RB1*, *STK11*, *THBS1*, *TIMP3*, *TP53*, *TP73*, *VHL,WT1*	Unsupervised hierarchical clustering of the 33 methylated genes clustered PNETs into three groups, with increased numbers of TSG gene methylation associated with poorer prognosis. *LINE-1* hypomethylation was also associated with poorer overall survival
Liu *et al.* (2014)	350 PNETs: 140 INS, 144 NF, 39 functional NOS	DHPLC	*INA* (56%) – analysed in 25 patient PNET samples only.	Cell lines treated with decitabine to look for protein re-expression
Choi *et al.* (2007)	11 PNETs with matched controls	PSQ	*LINE-1*, *Alu* hypomethylation	Relative hypomethylation of *LINE-1* associated with metastasis and *RASSF1A* methylation. *Alu* hypomethylation correlated with *MGMT* methylation
Cros *et al.* (2016)	43 PNETs: (5 MEN1)	PSQ, IHC, and MSI	*MGMT*	Low MGMT expression was associated with longer progression-free survival and response to temozolomide therapy
Dejeux *et al.* (2009)	32 PNETs: 12 INS, 20 NF	PSQ, mRNA, and IHC	IGF2-H19 locus	Increased expression (IHC and mRNA) of IGF2 in insulinomas and increased methylation of the DMR2 locus. Decreased DMR methylation associated with increasing tumour grade.
Evans *et al.* (2022)	27 PNETs	PSQ	*SSTR2*	Promoter methylation of *SSTR2* is higher in PNETs compared to non-NET tissue and is inversely correlated with IHC SSTR2 staining. Guadecitabine increases SSTR2 expression in NET cell lines (and xenograft mouse model)
Stricker *et al.* (2012a)	15 PNETs with matched controls	PSQ	*LINE-1* hypomethylation	LINE-1 hypomethylation was associated with lymph node metastasis and grade 1 vs 2 PNETs
Zhang *et al.* (2020)	14 PNETs: 7 ACTH, 7 NF	PSQ and IHC	*POMC*	The *POMC* promoter was hypomethylated in ACTH-PNETs compared to NF-PNETs and normal pancreas.

^a^All genes had hypermethylation in >10% of MEN1-related tumours analysed. 450K, Infinium HumanMethylation450 BeadChip; 5-aza-C, azacitadine; A-D-M, ATRX-DAXX-MEN1; APAF1, apoptotic peptidase-activating factor 1; APC, APC regulator of WNT signalling pathway; ATAC-seq, assay for transposase-accessible chromatin with high-throughput sequencing; ATM, ATM serine/threonine Kinase; ATRX, ATRX chromatin remodeler; BCL2, BCL2 apoptosis regulator; BRCA1, BRCA1 DNA repair associated; BRCA2, BRCA2 DNA repair associated, CACNA1G, T-type calcium channel; CADM1, cell adhesion molecule 1; CASP8, caspase 8; CD44, CD44 molecule (Indian Blood Group); CDH1, E-cadherin; CDH13, cadherin 13; CDKN1B, cyclin-dependent kinase inhibitor 1B; CDKN2A(p14, p16), cyclin-dependent kinase inhibitor 2A; CDKN2B, cyclin-dependent kinase inhibitor 2B; CHFR, checkpoint with forkhead and ring-finger domains; CNA, copy number alterations; COBRA, combined bisulfite restriction analysis; COX2, cyclooxygenase 2; DAPK1, death-associated protein kinase 1; DAXX, death domain-associated protein; DHPLC, denaturing high-performance liquid chromatography; DMR, differentially methylated region; DNAJC15, DnaJ heat-shock protein family (Hsp40) member C15; EPIC, Infinium MethylationEPIC BeadChip; ER, oestrogen receptor; ESR1, oestrogen receptor 1; FHIT, fragile histidine triad diadenosine triphosphatase; GAS, gastrinoma; GATA4, GATA-binding protein 4; GATA5, GATA-binding protein 5; GLU, glucagonoma; GSTP1, glutathione S-transferase Pi 1; H19, H19 imprinted maternally expressed transcript; HIC-1, HIC ZBTB transcriptional repressor 1; hMLH1, human MutL homolog 1; 5hmC, 5’ hydroxymethylcytosine; HOPX, HOP homeobox; IGF2, insulin-like growth factor 2; IHC, immunohistochemistry; INA, internexin neuronal intermediate filament protein alpha/alpha-internexin; INS, insulinoma; Killin, KLLN, p53-regulated DNA replication inhibitor; LINE-1, long interspersed nuclear element 1; LOH, loss of heterozygosity; MEN1, multiple endocrine neoplasia type-1; MGMT, O6-methyl-guanine methyltransferase; MINT1/ABPBA1, amyloid beta precursor protein-binding family A member 1; MINT2/ABPBA2, amyloid beta precursor protein-binding family A member 2; MINT25, MINT27, and MINT31, methylated In tumour; mRNA, messenger ribonucleic acid; MS-MLPA, methylated-specific multiplex ligation-dependent probe amplification; MSH6, MutS homolog 6; MSI, microsatellite instability; MSP, methylation-specific-PCR; MUS81, MUS81 structure-specific endonuclease subunit; NF, non-functioning; NF1, neurofibromin 1; NOS, not otherwise specified; NS, not significant; NTRK1, neurotrophic receptor tyrosine kinase 1; PAX5, paired box 5; PAX6, paired box 6; PCCA, propionyl-CoA carboxylase subunit alpha; PDX1, pancreatic and duodenal homeobox 1; PNEC, pancreatic neuroendocrine carcinoma; PNEN, pancreatic neuroendocrine neoplasm; PNET, pancreatic neuroendocrine tumour; POMC, proopiomelanocortin; PSQ, pyrosequencing; PYCARD, PYD and CARD domain containing; Q-MSP, quantitative-MSP; RB1, RB transcriptional corepressor 1; RARb, retinoic acid receptor beta 2; RARRES1, retinoic acid receptor responder 1; RASSF1A, Ras association domain family member 1; REN, renin producing; SFRP1, secreted frizzled related protein 1; SOM, somatostatinoma; SSTR2, STK11, somatostatin receptor 2; serine/threonine kinase 11; TERT, telomerase reverse transcriptase; THBS1, thrombospondin 1; TIMP3, TIMP metallopeptidase inhibitor 3; TP53, tumour protein P53; TP73, tumour protein P73, TSG, tumour-suppressor gene; TWIST1, twist family BHLH transcription factor 1; VHL, Von Hippel–Lindau tumour suppressor; VIP, VIPoma; WES, whole-exome sequencing; WGS, whole-genome sequencing; WTI, WT1 transcription factor.

**Table 3:** Global DNA methylation and hydroxymethylation studies of human PNETs.

Study	PNETs	Investigation	Genes investigated (proportion methylated (%))	Other features
**Global methylation assessment**				
Marinoni *et al.* (2017)	167 PNETs	Episeeker quantification, IHC, MSP and PSQ	Global and *LINE-1* methylation	DAXX/ATRX-negative tumours and patients with MEN1 mutations were not associated with *LINE-1* hypomethylation
Tirosh *et al.* (2019)	33 PNETs (9 sporadic, 10 MEN1, and 10 VHL)	EPIC	Methylation assessment of >850,000 CpGs across the human genome	Reported loss of PHLDA3 as being an important gene involved upstream of the Akt pathway.
Di Domenico *et al.* (2020)	125 PNETs	450K	Compared DNA methylome of PNETs to sorted normal alpha and beta cells	Stratified PNETs by DNA methylation signatures which improved patient stratification which correlated with disease-free survival
Chan *et al.* (2018)	64 PNETs (32 PNETs methylome investigated)	450K	Compared the DNA methylome in PNETs grouped by A-D-M mutant vs wildtype	*A-D-M* mutant PNET had a similar methylation profile to that of an alpha cell, with high ARX and low PDX1
Boons *et al.* (2020)	83 PNETs (26 methylome investigated)	EPIC	Compared to DNA methylome of five normal islet cells (two alpha cells and three beta cells)	PNETs were categorised into alpha- or beta-like tumours based on methylation signatures
Lakis *et al.* (2021)	84 PNETs	450K	Compared to DNA methylome of 11 normal adjacent pancreata	PNETs were categorised into three subgroups T1: functional tumours with *A-D-M* wildtype (similar to beta cell), T2: *A-D-M* mutant, and T3: *MEN1* mutations (similar to alpha cells)
Simon *et al.* (2022)	57 PNENs (43 PNETs and 14 PNECs)	EPIC	Compared the DNA methylome of PNETs and PNECs to that of cell type signatures of alpha, beta, acinar, and ductal adult cells.	PNEC had similar methylomes to exocrine tissue
Yachida *et al.* (2022)	PNENs (48 PNETs and 18 PNECs)	EPIC, WGS, WES, ATAC-seq	Compared the DNA methylome of PNETs of PNECs	*DAXX* hypermethylation ~ all PNETs. PNECs clustered into ‘ductal’ or ‘acinar’ types. PNETs clustered into (i) *MEN1* alterations with *RASSF1A*, *PDX1,* and *CDX2* promoter hypermethylation and (ii) hypomethylation group including: *HNF4A*, *MGMT,* and *TERT*.
**Hydroxymethylation assessment**				
Sharma *et al.* (2022)	60 PNETs	IHC	5hmC staining of formalin-fixed paraffin-embedded slides	Loss of 5hmC was associated with metastatic disease

450K, Infinium HumanMethylation450 BeadChip; ARX, Aristaless-related homeobox; A-D-M, ATRX-DAXX-MEN1; ATAC-seq, assay for transposase-accessible chromatin with high-throughput sequencing; ATRX, ATRX chromatin remodeler; CDKN2A(p14, p16); cyclin-dependent kinase inhibitor 2A; CDX2, caudal-type homeobox protein 2; DAXX, death domain-associated protein; EPIC, Infinium MethylationEPIC BeadChip; HNF4A, hepatocyte nuclear factor 4 alpha; IHC, immunohistochemistry; LINE-1, long interspersed nuclear element 1; MEN1, multiple endocrine neoplasia type 1; MGMT, O6-methyl–guanine methyltransferase; mRNA, messenger ribonucleic acid; MSP, methylation-specific PCR, NF, non-functioning; PNEC, pancreatic neuroendocrine carcinoma; PNEN, pancreatic neuroendocrine neoplasm; PNET, pancreatic neuroendocrine tumour; PSQ, pyrosequencing; RASSF1A, Ras association domain family member 1; TERT, telomerase reverse transcriptase; VHL, Von Hippel–Lindau tumour suppressor; WES, whole-exome sequencing; WGS, whole genome sequencing.

Approximately 30% of these 24 studies (7/24), which used a targeted hypothesis-driven approach, have reported that the TSGs, Ras association domain family member 1 (*RASSF1)* and cyclin-dependent kinase inhibitor 2A (*CDKN2A)*, were found to be methylated in up to 83% (Dammann *et al.* 2003, House *et al.* 2003, Liu *et al.* 2005, Arnold *et al.* 2007, Malpeli *et al.* 2011, Stefanoli *et al.* 2014, Conemans *et al.* 2018) and 17–67% (Muscarella *et al.* 1998, Bartsch *et al.* 2000, Serrano *et al.* 2000, Chan *et al.* 2003, Dammann *et al.* 2003, House *et al.* 2003, Liu *et al.* 2005, Arnold *et al.* 2007, Stefanoli *et al.* 2014) in PNETs, respectively. The *RASSF1* gene has two promoters (A and C) and seven different transcripts (RASSF1A–G). RASSF1A is a ubiquitously expressed scaffold protein which interacts with many different pathways, including the Wnt and Hippo pathways (Papaspyropoulos *et al.* 2018). *CDKN2A* encodes for two separate proteins p14 and p16 (INK4a) and is involved in cell cycle regulation, and a loss of function of CDKN2A is associated with cancer (Ruas & Peters 1998). One study investigated the Pleckstrin homology-like domain family A member 3 (*PHLDA3)* gene and reported loss of PHLDA3 expression *via* loss of heterozygosity and promoter methylation, which was seen in up to 72% (36/50) of PNETs, and this is comparable to that seen with menin loss of expression (60–67%). PHLDA3 is a tumour suppressor which acts by competing with Akt and inhibiting its interaction and subsequent activation with membrane lipids. Therefore, loss of PHLDA3 leads to increased Akt activation and subsequently increased signalling through the phosphatidylinositol-3-kinase/Akt/mTOR (PI3K/Akt/mTOR) pathway, which is commonly upregulated in PNETs. PHLDA3 knockout also leads to beta cell proliferation, as illustrated by studies in a PHLDA3^–/–^ knockout mouse model (Ohki *et al.* 2014). Increased somatostatin receptor 2 (*SSTR2)* methylation was reported in 27 human PNET samples compared to non-NET tissue, and this was inversely correlated to SSTR2 expression by immunohistochemistry (Evans *et al.* 2022*a*). Recently, DAXX was reported to be hypermethylated in almost all PNETs (Yachida *et al.* 2022). Overall, half of these studies have investigated protein and/or mRNA expression with their relationships to promoter and enhancer methylation and have reported a variable association between gene methylation and expression (Bartsch *et al.* 2000, Wild *et al.* 2003, Dejeux *et al.* 2009, Malpeli *et al.* 2011, Ohki *et al.* 2014, Schmitt *et al.* 2014, Cros *et al.* 2016, Ushiku *et al.* 2016, Marinoni *et al.* 2017, Tirosh *et al.* 2019, Zhang *et al.* 2020, Ban *et al.* 2022, Evans *et al.* 2022*a*, Yachida *et al.* 2022).

Five of these studies have investigated DNA hypomethylation in PNETs, using LINE-1 and *Alu* hypomethylation as a surrogate for global DNA methylation (Choi *et al.* 2007, Stricker *et al.* 2012, Stefanoli *et al.* 2014, Marinoni *et al.* 2017, Yachida *et al.* 2022). LINE-1, the most abundant LINE, is located non-randomly in GC-poor regions of DNA, approximately 6000 kb long, and encodes for two proteins which catalyse retro-transposition, i.e. the ability to ‘copy and paste’ itself (i.e. LINE-1) into other sections of DNA (Choi *et al.* 2007). Multiple copies or fragments of LINE-1 are present throughout the human genome and are usually transcriptionally silenced by either truncating mutations within the 5’UTR and/or promoter region or by methylation (Carnell & Goodman 2003, Hancks & Kazazian 2016, Sanchez-Luque *et al.* 2019). Global DNA hypomethylation and LINE-1 promoter hypomethylation and subsequent transcription leads to genetic instability, increases the mutation rate, and has been associated with different cancers, e.g. breast, colon, lung, head and neck, bladder, liver, prostate, oesophagus, stomach (Chalitchagorn *et al.* 2004), and PNETs (Chen *et al.* 1998, Takai *et al.* 2000, Choi *et al.* 2007, Stricker *et al.* 2012, Stefanoli *et al.* 2014, Marinoni *et al.* 2017). *Alu* elements are repetitive elements ~280 base pairs long, and are usually heavily methylated in normal pancreatic tissue and are hypomethylated in PNETs. *Alu* methylation was significantly inversely correlated with MGMT promoter methylation, with low levels of *Alu* methylation found in patients with well-differentiated PNETs and carcinoid tumours, who had MGMT methylation (Choi *et al.* 2007). In addition to LINE-1 and *Alu* hypomethylation, telomerase reverse transcriptase, *MGMT* and hepatocyte nuclear factor 4 alpha are hypomethylated in a subset of PNETs which tended to harbour MEN1 alterations and greater promoter hypermethylation in *RASSF1*, pancreatic and duodenal homeobox 1 (*PDX1*), and caudal type homebox 2 (Yachida *et al.* 2022). One study investigated alterations in enhancer regions and reported that the enhancer for the protein tyrosine phosphatase receptor type N2 (*PTPRN2)* gene was hypomethylated in all PNET subgroups and was associated with increased PTPRN2 transcription (Tirosh *et al.* 2019). PTPRN2 is highly expressed in islet cells and is upregulated in other cancers, including breast and hepatocellular cancer (Shen *et al.* 2015, Sengelaub *et al.* 2016). Of note, most studies that have reported on the DNA methylome in PNETs have focused on 5mC, with only one study reporting loss of global 5hmC to be associated with tumourigenesis (Sharma *et al.* 2022).

### DNA methylation in hereditary-associated vs sporadic PNETs

Two studies have compared DNA methylation in patients with sporadic PNETs to that in patients with PNETs associated with hereditary syndromes (MEN1 and VHL) (Conemans *et al.* 2018, Tirosh *et al.* 2019). One study compared cumulative methylation indices and gene-specific methylation levels in 56 TSGs in 95 PNETs (61 MEN1 vs 34 sporadic) and reported that overall DNA methylation levels were comparable and that DNA methylation was increased in larger tumours and in metastatic disease (Conemans *et al.* 2018). However, another study compared global methylation levels in 30 non-functional PNETs (10 sporadic and 10 each from patients with MEN1 and VHL) and four normal islet samples, using the Illumina MethylationEPIC array, and reported significantly increased DNA methylation in patients with MEN1 than that in sporadic and VHL-associated PNETs. This global hypermethylation seen in PNETs from patients with MEN1 was also seen in two *MEN1*-knockout mouse models, (*Pdx1-Cre: MEN1 floxed/floxed* (pancreatic) and *P*th*-Cre:Men1 floxed/floxed* (parathyroid)). The observed hypermethylation in the *P*th*-Cre:Men1 floxed/floxed* was consistent with that reported in 12 patients with MEN1-associated hyperparathyroidism, when compared to 13 sporadic parathyroid adenomas, 4 parathyroid carcinomas, and 9 normal parathyroids, using *Hpa*II tiny fragment enrichment by ligation-mediated PCR (HELP), which specifically measures global 5mC marks (Kinney *et al.* 2011, Yuan *et al.* 2016). These reported differences in MEN1-associated DNA methylation levels may, however, partly be explained by study methodology, as two studies assessed global DNA methylation levels (methylationEPIC array and HELP, respectively) whereas, one study examined 56 specific TSGs, by MS-MLPA (Nygren *et al.* 2005, Conemans *et al.* 2018, Tirosh *et al.* 2019).

Patients with MEN1 syndrome mainly develop tumours in endocrine organs, including the pituitary, pancreas and parathyroid glands; however, it is unclear why menin loss specifically increases the risk of tumours in these particular organs and not others. Different DNA methylation patterns have been reported *in vivo* in mouse endocrine vs exocrine pancreatic tissue in the *Pdx1-Cre: MEN1 floxed/floxed*, menin-knockout mouse model (Yuan *et al.* 2016). The gene RB-binding protein 5, histone lysine methyltransferase complex subunit (*RBBP5*), which encodes for the RbBP5 protein, one of the subunits involved in the WRAD complex (WDR5, RbBP5, Ash2L and Dpy30) that is required by KMT2A/B for H3K4 methylation (Mittal *et al.* 2018), binds to the DNMT1 promoter in both endocrine and exocrine tissue, however, increased DNMT1 expression is only observed in the endocrine pancreas (Yuan *et al.* 2016), and this may be associated with (or due to) menin loss that can lead to increased DNA methylation *via* increased DNMT1 expression (Fig. 2). Pathways enriched for hypermethylated genes in tumours developing in MEN1-knockout mice included those involved in the Wnt/beta-catenin pathway, with increased beta-catenin levels secondary to loss of Sox-regulatory proteins by promoter methylation (Yuan *et al.* 2016). In PNETs from patients with MEN1, promoter methylation in two genes has been reported: cell division cycle associated 7 like and RNA-binding motif protein 47 (Tirosh *et al.* 2019), with aberrant expression reported in other malignancies, including paediatric pineal germinomas and colorectal cancer (Perez-Ramirez *et al.* 2017, Rokavec *et al.* 2017). Findings from these studies may be explained by menin-mediating H3K4 methylation, an active histone mark, which may protect DNA from methylation (Cedar & Bergman 2009), or alternatively, menin loss may lead to increased global DNA methylation and gene specific TSG methylation (Fig. 2) (Iyer & Agarwal 2018). Loss of menin expression in both endocrine and exocrine cells, as occurring in the *Pdx1-Cre: MEN1 floxed/floxed* mouse model, was not observed to alter DNMT1 expression in the exocrine pancreas, thereby suggesting that menin is important in maintaining the DNA methylome in endocrine cells, and this may provide an explanation for the predominant development of tumours in endocrine organs in patients with MEN1.

## Translational utility of PNET DNA methylation

### DNA methylation to define and stratify PNETs

There are five types of endocrine cells within the islets of Langerhans, which comprise ~54% beta (insulin-secreting), ~35% alpha (glucagon-secreting), and ~11% delta (somatostatin-secreting), with a small number of gamma/pancreatic polypeptide (PP-secreting) cells (Lawlor *et al.* 2017). Epigenetic signatures have been used to stratify PNETs into distinct categories, using either enhancer maps (histones marks) (Cejas *et al.* 2019) or DNA methylation. There have been five studies comparing the DNA methylation signatures of PNETs to either normal pancreatic islet methylomes or PNECs to stratify these into different groups (Chan *et al.* 2018, Boons *et al.* 2020, Di Domenico *et al.* 2020, Lakis *et al.* 2021, Yachida *et al.* 2022). Two studies which stratified PNETs from PNECs reported that PNECs have a similar methylation profile to exocrine pancreatic tissue (Simon *et al.* 2022, Yachida *et al.* 2022). One of these studies used multiomic data to further stratify PNECs into ‘ductal’ (retinoblastoma-associated protein (RB1) protein loss, tumour protein 53 (*TP53)* mutations, and a CpG island methylator phenotype (CIMP) phenotype) and ‘acinar’ (*CDKN2A* alterations, deletions or promoter hypermethylation, and WNT signalling alterations) subtypes (Yachida *et al.* 2022). These studies provide further evidence that PNECs are a distinct biological entity when compared to PNETs and highlight the importance of accurate tumour diagnosis to ensure that patients receive the appropriate therapies. The DNA methylome has been reported in the two most common islet cell types alpha and beta cells, each with their own unique methylation signature (Boons *et al.* 2020, Di Domenico *et al.* 2020, Simon *et al.* 2022). The methylation signature of insulinomas (pancreatic islet tumours which secrete excess insulin) closely aligns with that of a normal beta cell methylation profile, consistent with its cell of origin (Di Domenico *et al.* 2020). Insulinomas account for 10% of PNETs seen in patients with MEN1 (Larsson *et al.* 1988, Thakker *et al.* 2012); however, sporadic insulinomas (and other functional PNETs) (Lakis *et al.* 2021) are frequently wildtype for *MEN1*, *ATRX,* or *DAXX* (Cao *et al.* 2013, Di Domenico *et al.* 2020, Lakis *et al.* 2021) and express menin (Arnold *et al.* 2007). The most common reported genetic driver of insulinomas (seen in up to 30%) involves the amino acid mutation Thr372Arg in Yin Yang 1(*YY1*) which acts through the mTOR pathway (Cao *et al.* 2013, Hong *et al.* 2020). *YY1* is an evolutionary conserved ubiquitous protein involved in transcriptional activation or repression by recruitment of histone methyltransferases and plays a crucial role in ensuring *LINE-1* methylation (Seto *et al.* 1991, Rezai-Zadeh *et al.* 2003, Sanchez-Luque *et al.* 2019). PNETs which share a similar methylation signature to normal alpha-cells (high ARX and low PDX1) tend to harbour only *MEN1* mutations or have lost menin expression (Di Domenico *et al.* 2020, Lakis *et al.* 2021, Yachida *et al.* 2022). However, the majority of PNETs display a methylation signature somewhere between alpha and beta cells, with approximately 70% of these harbouring mutations in *MEN1* and/or *ATRX* and *DAXX* (Di Domenico *et al.* 2020). PNETs have also been stratified into A-D-M (ATRX/DAXX/MEN1) mutant vs wildtype, with A-D-M mutant PNETs tending to display similar methylation features to that of an alpha cell, whereas, A-D-M wildtype PNETs were more heterogenous with a subset showing similar profiles to beta-cells (Chan *et al.* 2018). DNA methylation patterns have also been used to classify NETs by location and to determine the origin of NETs of unknown primary (How-Kit *et al.* 2015, Hackeng *et al.* 2021).

### Clinical outcomes in PNETs

In PNETs, the majority of studies have looked at the presence or absence of a specific TSG and have either correlated this with overall survival (Ohki *et al.* 2014, Schmitt *et al.* 2014, Stefanoli *et al.* 2014, Ushiku *et al.* 2016), with increased tumour grade (Dejeux *et al.* 2009), or with the presence of metastasis (Dammann *et al.* 2003, Wild *et al.* 2003, Choi *et al.* 2007). *RASSF1* and *CDKN2A* were found to be methylated in 100% and 40% of patients with metastatic disease vs 71% and 0% without metastases (Dammann *et al.* 2003). Multivariate analysis, controlling for clinical factors including tumour grade, size, and stage, reported that *CDKN2A* (House *et al.* 2003) and *MGMT* (Schmitt *et al.* 2014, Ban *et al.* 2022) methylation, but not *RASSF1* (House *et al.* 2003), were associated with mortality. Other studies have reported that low MGMT expression and promoter hypomethylation predict response to temozolomide chemotherapy (Cros *et al.* 2016, Campana *et al.* 2018). However, a low MGMT expression was also observed in a high proportion of patients (75% (6/8)) with PNETs who did not respond to temozolomide, and it seems that a low MGMT expression has a high sensitivity, but low specificity to predict temozolomide response (Cros *et al.* 2016). TIMP metallopeptidase inhibitor 3 methylation has also been associated with metastatic disease, with strong staining seen in normal islets and decreased expression in 55% of patients with PNETs on univariate analysis (Wild *et al.* 2003). CIMP positivity, defined as multiple methylated TSGs (although there is no clear cutoff to determine CIMP positivity), has been associated with multiple cancers, including those of the colorectum, lung and prostate, and gliomas (Toyota & Issa 1999, Yates & Boeva 2022). Investigation of the CIMP-positive phenotype in PNETs has indicated that it is associated with a poorer overall survival (Arnold *et al.* 2007, Stefanoli *et al.* 2014). In addition, progressive LINE-1 hypomethylation has been associated with increased mortality (Stefanoli *et al.* 2014), lymph node metastases (Choi *et al.* 2007), and tumour grade (Choi *et al.* 2007, Stricker *et al.* 2012). No study has reported a difference in PNET methylation between sexes (Choi *et al.* 2007, Campana *et al.* 2018, Boons *et al.* 2020, Ban *et al.* 2022), although loss of global 5hmC was reported to be associated with tumourigenesis and to correlate with distant metastasis and female gender in a multivariate analysis of 60 well-differentiated PNETs (Sharma *et al.* 2022). The DNA methylome has also been used to cluster PNETs into different prognostic categories (Chan *et al.* 2018, Boons *et al.* 2020, Di Domenico *et al.* 2020, Simon *et al.* 2022). Studies clustering PNETs into two categories (A-D-M/ATRX-DAXX-MEN1) mutant vs wildtype have reported that the A-D-M mutant category (ARX positive and PDX1 negative) had an overall worse prognosis, when compared to A-D-M wildtype (Chan *et al.* 2018) and PNETs with a beta-like cell methylation signature (Boons *et al.* 2020, Lakis *et al.* 2021). Other studies clustering PNETs into alpha-like, beta-like, or intermediate tumours have reported that intermediate tumours tend to be less differentiated and of higher grade when compared to the alpha-like or beta-like PNETs and that using the DNA methylome to stratify PNETs into these three groups more accurately predicted disease-free survival when compared to the analysis of transcription factor expression, by immunohistochemistry for alpha-cell specific (ARX), beta-cell specific (PDX1), or intermediate (DAXX/ATRX) alone (Di Domenico *et al.* 2020). Similar results were found in a large international cohort of NETs, including 561 primary NF-PNETs and 107 metastatic NF-PNETs, which reported that ARX or PDX1 expression did not independently predict relapse-free survival (RFS), whereas ATRX/DAXX loss and alternative lengthening of telomeres (ALT) status were both independent predictors of RFS (Hackeng *et al.* 2022). Using DNA methylation to compare PNETs to their differentiated non-cancerous counterparts (i.e. alpha and beta cells) has been used to prognostically stratify patients, and utilising this methodology appears to be more discriminative in terms of predicting prognosis. Thus, studies have reported that beta cell phenotypes tend to have a better prognosis; however, it is important to note that tumours secreting hormones (e.g. insulin) tend to be detected at earlier stages than non-secreting (i.e. non-functioning) tumours, and this may be a confounding factor if it is the cell of origin that determines tumour aggressiveness. Given that the majority of insulinomas are indolent/typical (i.e. non-metastatic), epigenetic signatures comparing indolent vs aggressive (i.e. metastatic) insulinomas have not been investigated, likely due to the rare nature of metastatic insulinomas. One recent study reported ARX expression in all aggressive compared to indolent insulinomas and suggested that these aggressive tumours originated from an alpha-like cell and inappropriately gained insulin secretion (Hackeng *et al.* 2020). Another example of an inappropriate gain in secretory properties of PNETs is ectopic adrenocorticotrophin (ACTH)-secreting PNETs. One study reported lower pro-opiomelanocortin methylation of seven ACTH-PNETs when compared to seven clinically NF-PNETs. The 1-year survival for patients with ACTH-secreting PNETs was 57% (Zhang *et al.* 2020). This poor overall survival seen in patients with ACTH-secreting PNETs may be explained by the high morbidity associated with uncontrolled hypercortisolism (Kamp *et al.* 2016), and by the fact that islet cells do not normally secrete ACTH and therefore, such PNETs may harbour other epigenetic and/or genetic abnormalities which carry a poorer prognosis.

### Therapeutic targeting of aberrant DNA methylation

The most common class of anti-cancer drugs used to alter the DNA methylome are inhibitors of DNMTs. DNMT inhibitors (DNMTi) may show efficacy by improving the cancer phenotype, directly (through the re-expression of the apoptotic pathway and/or cell cycle inhibitors) or indirectly by the re-expression of receptors or transcription factors which may help to overcome drug resistance, as seen with other types of chemotherapy. Azacitadine and its derivative decitabine (5–2’-deoxycytidine; first-generation DNMTi) and guadecitabine (second generation) are incorporated into replicating DNA in place of cytidine. DNMTs methylate this incorporated analogue but are unable to dissociate from DNA and are subsequently degraded, thereby leading to overall DNMT depletion and subsequent loss of DNA methylation (Hu *et al.* 2021). These drugs have been assessed using the human PNET cell lines (BON-1 (derived from a lymph node with metastatic insulinoma) (Townsend *et al.* 1993), QGP-1 (derived from a pancreatic somatostatinoma) (Kaku *et al.* 1980), and/or CM (derived from ascitic fluid from a metastatic insulinoma)) (Baroni *et al.* 1999) and were found to increase the expression of *RASSF1A* (Malpeli *et al.* 2011) and *SSTR2 in vitro* and in an *in vivo* (mouse xenograft) model (Taelman *et al.* 2016, Evans *et al.* 2022*a*). Despite being used clinically for other malignancies, e.g. haematological malignancies, this class of drug has only been used in one small clinical trial of nine patients with NETs, including two patients with PNETs, who exhibited low baseline SSTR2 expression on 68Ga-DOTATE imaging (Refardt *et al.* 2022). In this study, hydralazine, a common anti-hypertensive medication, which in this case was utilised for its DNMTi properties, was administered daily in combination with an HDAC inhibitor (valproic acid, a common anti-epileptic medication), with the aim of upregulating SSTR2. Despite previous *in vitro* and *in vivo* (mouse xenograft) models reporting upregulation of SSTR2 (Taelman *et al.* 2016, Evans *et al.* 2022*a*) using decitabine or guadecitabine, hydralazine treatment was unable to upregulate SSTR2 in either BON-1 or human PNETs (Refardt *et al.* 2022). The current cell lines used to investigate DNA methylation in PNETs tend to be highly proliferative and harbour genetic mutations similar to those found in PNECs (e.g. KRAS proto-oncogene GTPase (*KRAS*) mutations in (*KRAS*) mutations found in the QGP-1 cell line) (Kaku *et al.* 1980), and therefore the direct translatability of results from using these cells lines *in vitro* to the less proliferative human PNETs, which do not tend to harbour these genetic mutations *in vivo,* is unclear. Temozolomide therapy, with and without capecitabine, has been used in the treatment of neuroendocrine tumours including PNETs. Temozolomide (a type of chemotherapy drug), which works as an alkylating agent by forming adducts on the O6 and N7 positions of guanine and without MGMT to remove these, leads to cell death (Campana *et al.* 2018). Temozolomide-based chemotherapy has been reported in two clinical studies in a total of 138 PNETs, which reported low MGMT expression as a strong predictive factor for longer progression-free and overall survival (Cros *et al.* 2016, Campana *et al.* 2018). Somatostatin analogues (SSAs) are the most commonly used medical therapy for patients with PNETs, with different SSA compounds having different affinities for the somatostatin receptor subtypes (SSTR_1–5_). It has been reported that response to SSA treatment does not solely depend on the tumour receptor subtype expression and that other tumour factors modulate its treatment effect, for example, the natural antisense transcript of SSTR5-ASI (Pedraza-Arevalo *et al.* 2022).

## Conclusions and future perspectives

Studies examining PNET DNA methylation levels have largely focussed on a specific subset of TSGs using MSPs, and only a subset of these have correlated protein expression with clinical outcomes. However, given the increasing availability of methylation array-based technologies in conjunction with RNA-seq, further studies looking at how changes in the DNA methylome affect cellular phenotype are likely to become mainstream. Given that 5hmC has been shown to be associated with gene transcription and protects CpG sites from methylation (5mC), newer techniques which are able to separate the specific 5’ cytosine modifications and correlate these with gene expression are needed. Current therapies (e.g. DNMTi) used *in vitro* have shown efficacy in PNET cell lines via re-expressing TSGs, but these cell lines tend to be highly proliferative and not representative of the more common relatively indolent PNETs, and studies in more representative cell lines and models are required. Investigating the PNET DNA methylome and using this to determine its cell of origin (i.e. alpha/beta/indeterminate cell like) will help progress in PNET research by clustering tumour subtypes epigenetically, which may help to prognostically stratify patients and to guide which patients may benefit from targeted epigenetic therapy. As yet, there have been no reported studies looking at changing the DNA methylome to improve cellular phenotype (and response to other anti-cancer agents in combination), which is likely to be an important way forward, particularly given that PNETs displaying similar phenotypes to normal alpha and beta cells have a more favourable prognosis.

## Declaration of interest

There is no conflict of interest that could be perceived as prejudicing the impartiality of this review.

## Funding

This work was supported by Cancer Research UKhttp://dx.doi.org/10.13039/501100000289 (CRUK) grant number C2195/A28699, through a CRUK Oxford Centre Clinical Research Training Fellowship (KE); National Institute for Health Researchhttp://dx.doi.org/10.13039/100005622 (NIHR) Senior Investigator Award (RVT); and NIHR Oxford Biomedical Research Centrehttp://dx.doi.org/10.13039/501100013373 Programme (KL,RVT).

## Author contributions statement

KE wrote the manuscript; RVT and KL edited the manuscript.
